# Dopamine Related Genes Differentially Affect Declarative Long-Term Memory in Healthy Humans

**DOI:** 10.3389/fnbeh.2020.539725

**Published:** 2020-10-28

**Authors:** Carla Leukel, Dirk Schümann, Raffael Kalisch, Tobias Sommer, Nico Bunzeck

**Affiliations:** ^1^Department of Psychology, University of Lübeck, Lübeck, Germany; ^2^Department of Systems Neuroscience, University Medical Center Hamburg-Eppendorf, Hamburg, Germany; ^3^Neuroimaging Center (NIC), Focus Program Translational Neuroscience, Johannes Gutenberg University Medical Center, Mainz, Germany; ^4^Leibniz Institute for Resilience Research (LIR), Mainz, Germany; ^5^Center of Brain, Behavior and Metabolism (CBBM), University of Lübeck, Lübeck, Germany

**Keywords:** reward, long-term memory, polymorphism, dopamine, motivation

## Abstract

In humans, monetary reward can promote behavioral performance including response times, accuracy, and subsequent recognition memory. Recent studies have shown that the dopaminergic system plays an essential role here, but the link to interindividual differences remains unclear. To further investigate this issue, we focused on previously described polymorphisms of genes affecting dopaminergic neurotransmission: DAT1 40 base pair (bp), DAT1 30 bp, DRD4 48 bp, and cannabinoid receptor type 1 (CNR1). Specifically, 669 healthy humans participated in a delayed recognition memory paradigm on two consecutive days. On the first day, male vs. female faces served as cues predicting an immediate monetary reward upon correct button presses. Subsequently, participants performed a remember/know recognition memory task on the same day and 1 day later. As predicted, reward increased accuracy and accelerated response times, which were modulated by DAT 30 bp. However, reward did not promote subsequent recognition memory performance and there was no interaction with any genotype tested here. Importantly, there were differential effects of genotype on declarative long-term memory independent of reward: (a) DAT1 40 bp was linked to the quality of memory with a more pronounced difference between recollection and familiarity in the heterozygous and homozygous 10-R as compared to homozygous 9-R; (b) DAT1 30 bp was linked to memory decay, which was most pronounced in homozygous 4-R; (c) DRD4 48 bp was linked to overall recognition memory with higher performance in the short allele group; and (d) CNR1 was linked to overall memory with reduced performance in the homozygous short group. These findings give new insights into how polymorphisms, which are related to dopaminergic neuromodulation, differentially affect long-term recognition memory performance.

## Introduction

The dopaminergic system serves several, yet interconnected, functions. On the one hand, a functional loop between the medial temporal lobe and midbrain dopamine (DA) neurons drives the encoding of novel information into long-term memory (Lisman and Grace, 2005; Bunzeck and Düzel, 2006; Düzel et al., 2009; Bunzeck et al., 2014). On the other hand, DA neurons are known to be critical for reward processing (Schultz et al., 1997; Fiorillo et al., 2003; Tobler et al., 2005). Therefore, it is not surprising that reward motivation not only accelerates response times (Knutson et al., 2001; Bayer et al., 2013; Steiger and Bunzeck, 2017) and enhances physical effort (Pessiglione et al., 2007), but also improves subsequent long-term memory (Wittmann et al., 2005; Adcock et al., 2006). Evidence for a link between reward processing and invigoration of behavior through dopaminergic neuromodulation comes from computational models and empirical studies in animals and humans (Niv et al., 2007; Guitart-Masip et al., 2011; Dayan, 2012; Beierholm et al., 2013; Steiger and Bunzeck, 2017). Importantly, specific genes that affect dopaminergic neuromodulation have also been identified; yet the link between genetic polymorphisms and reward-dependent long-term memory remains less clear.

In a typical reward-dependent long-term memory task, a cue predicts monetary rewards for a given behavior, for instance, correct category judgments. The to-be-learned information, such as an image, often follows the cue, or it is a cue itself (for instance the categories living vs. non-living indicate reward vs. no reward). Subsequently, the effect of reward can be tested with free recall (in the case of words), or with scores of recollection and familiarity based recognition memory. Dual-process models assume that recognition can be associated with specific details or associations of the encoding episode (i.e., recollection), or in the absence of such recollective experience (i.e., familiarity). Support for dual-process models (Yonelinas et al., 1996, 2010) comes from functional imaging studies suggesting that different regions of the medial temporal lobe are involved in recognition memory depending on task demands and type of information (Diana et al., 2007). In particular, while the hippocampus and posterior parahippocampal gyrus are closely associated with recollection, the anterior parahippocampal gyrus is more associated with familiarity (Diana et al., 2007).

While several studies reported a promoting effect of reward on behavior, some could not replicate it (Callan and Schweighofer, 2008; Ariely et al., 2009; Sharifian et al., 2017; Steiger and Bunzeck, 2017), and others have even reported detrimental effects of reward on behavior (Mobbs et al., 2009; Chib et al., 2012; Kuhbandner et al., 2016). At the psychological level, this has been related to over motivation or anxiety in the context of reward (Callan and Schweighofer, 2008; Mobbs et al., 2009). However, interindividual differences in cognitive performance could also be due to genetic predisposition affecting dopaminergic neuromodulation. Indeed, genetic polymorphisms are known to affect the availability of neurotransmitters in the central nervous system, its retention time in the synaptic cleft through transporter availability, and receptor density (Breedlove et al., 2010). Therefore, it is reasonable to further investigate the relationship between genetic polymorphisms and performance in a reward-based declarative memory task.

Four* a priori* selected polymorphisms were investigated here: DAT1 40 base pair (bp), DAT1 30 bp, DRD4 48 bp, and cannabinoid receptor type 1 (CNR1). The human DA transporter (DAT1) exhibits several functional mutations, including a 40 bp variable number of tandem repeat (VNTR) polymorphism with the most common 9-repeat (9-R) and 10-repeat (10-R) alleles (Mitchell et al., 2000; Simsek et al., 2006). The 9-R allele of the DAT1 polymorphism has been associated with lower availability of DAT1 (Heinz et al., 2000; Cheon et al., 2005) possibly leading to increased striatal DA in the synaptic cleft (Schuck et al., 2013). Those findings are supported by *in vitro* (VanNess et al., 2005) and *in vivo* research (Brookes et al., 2007). Only a few studies found contrary results, showing that 10-R carriers had lower availability of DAT1 (Jacobsen et al., 2000; van Dyck et al., 2005). At the behavioral level, the 10-R allele has been associated with risk-taking, suggesting a possible link to reward processing (Mata et al., 2012), and worse memory performance in some studies (Simon et al., 2011; Li et al., 2013). In a reward-based memory task, 10-R homozygotes remembered rewarded pictures better compared to neutral ones, which could be linked to differences in hemodynamic activity within the striatum and hippocampus (Wittmann et al., 2013); since this study only included 24 participants, a replication of a link between DAT1 and reward-based learning appears reasonable. Finally, Raczka et al. (2011) could show that 9R carriers are quicker in learning fear extinction, possibly related to a higher dopaminergic prediction error signal when an expected aversive unconditioned stimulus does not occur in extinction (see also Kalisch et al., 2019). Taken together, research on the DAT1 40 bp polymorphism suggests a critical role not only for learning and memory but also for reward processing.

A second DAT1 polymorphism counts 30 bp (intron 8) ranging from four to nine repetitions (Brookes et al., 2006; Asherson et al., 2007). The most common alleles are the 5-repeat (5-R) and 6-repeat (6-R) allele, but initial research reported three repeat units less, resulting in 2- and 3-repeat alleles. Therefore, the literature mentions 2-repeat (2-R) and 5-repeat (5-R) alleles, respectively, as well as 3-repeat (3-R) allele and 6-repeat (6-R) alleles (Asherson et al., 2007). Here, the 5-R (2-R) and 6-R (3-R) genotypes are counted with one repetition less, meaning that the 2/5-R is counted as 4-repeat (4-R) and the 3/6-R as 5-repeat (5-R) allele. While the functional effects of the DAT1 30 bp polymorphisms remain to be investigated, Brookes et al. (2007) associated the 3-R allele with increased levels of DAT1 in post-mortem tissue. Moreover, DAT1 30 bp has been linked to attention deficit hyperactivity disorder (ADHD; Brookes et al., 2006) and addiction behavior (Guindalini et al., 2006; O’Gara et al., 2007; Smirnova et al., 2011). However, evidence for a link between DAT1 30 bp and long-term memory or reward processing, respectively, is scarce. Therefore, this polymorphism will also be investigated here.

Another polymorphism that affects the dopaminergic system is the 48 bp DA receptor D4 (DRD4) polymorphism ranging from 2 to 10-repeats (Lichter et al., 1993) with the 7-repeat (7-R) allele known to functionally enhance signal transduction (Asghari et al., 1995). At the behavioral level, 7-R allele carriers showed slower RT (Szekely et al., 2011) and a sample of children/adolescents with ADHD performed worse in working memory and executive tasks (Loo et al., 2008). However, in another study, adult 7-R allele carriers with ADHD performed better in a working memory task as compared to those without the 7-R allele (Boonstra et al., 2008) suggesting that the effects of DRD4 48 bp might depend on age and ADHD status (Altink et al., 2012). Additionally, the DRD4 48 bp 7-R allele polymorphism has been associated with novelty-seeking (Schinka et al., 2002), impulsivity (Eisenberg et al., 2007; Congdon et al., 2008; Varga et al., 2012), risk behavior (Dreber et al., 2009; Kuhnen and Chiao, 2009; Roussos et al., 2009) and ADHD (LaHoste et al., 1996; Faraone et al., 1999). Therefore, these findings suggest that DRD4 48 bp might also be related to reward-based long-term recognition memory.

Finally, the cannabinoid receptor type 1, a G-protein-coupled receptor (Matsuda et al., 1990), affects the dopaminergic system by influencing the release of DA (de Fonseca et al., 2001; Schandry, 2011). In this regard, an AAT sequence polymorphism has been identified ranging from 1 (Comings, 1998; Martínez-Gras et al., 2006) to 20 repeats (Dawson, 1995) encoded through the CNR1 gene. It has been most extensively studied in the clinical context, showing an association between the CNR1 polymorphism and Schizophrenia (Ujike et al., 2002; Martínez-Gras et al., 2006; Chavarría-Siles et al., 2008), addiction (Ponce et al., 2003; Ballon et al., 2006; Benyamina et al., 2011) and impulsivity (Ehlers et al., 2007). Additionally, CNR1 subgroups showed differences in procedural learning (Ruiz-Contreras et al., 2011) and a working memory task (Ruiz-Contreras et al., 2013). Taken together, the CNR1 polymorphism seems to be associated with reward-related behavior and differences in memory tasks and, therefore, will be further investigated in this study.

The present study investigated the effects of reward in the context of a two-day recognition memory paradigm. We expected reward to accelerate RT and increase accuracy during encoding, and to promote subsequent long-term recognition memory. Moreover, we investigated whether genotypes, that affect the dopaminergic system (DAT1 40 bp, the DAT1 30 bp, the DRD4 48 bp, and the CNR1), interact with those measures.

## Materials and Methods

### Participants

In total, 690 healthy humans participated in this study, which was part of a large behavioral test-battery, over 2 days. However, due to incomplete data in the behavioral tasks (see below) or lack of genotyping, 21 participants had to be excluded. Thus, the final sample included 669 subjects [mean age (±SD) = 24.37 (±3.39) years, 478 female]. The testing and data acquisition included several behavioral paradigms, questionnaires, and neuropsychological tests. Some of the results have already been reported elsewhere (Haaker et al., 2015, 2017; Lonsdorf et al., 2015; Schümann et al., 2018). On both days, participants arrived in the laboratory at 9 AM; on day 1, they provided a urine sample for drug testing as well as a saliva sample for genotyping and DNA extraction.

### Experimental Paradigm

**Figure 1:**
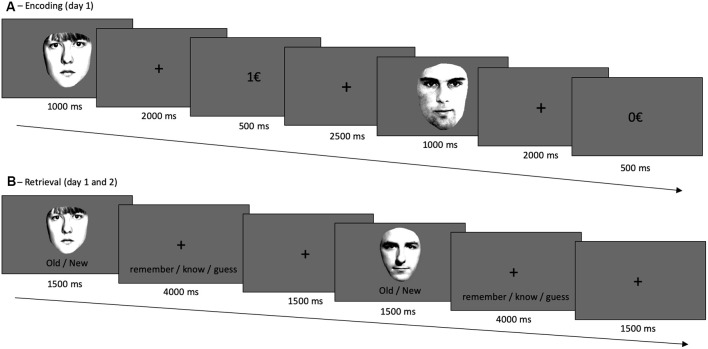
Experimental design. **(A)** During encoding, participants responded to male vs. female faces *via* button presses. One category was, upon correct categorization, rewarded; in this case female faces. **(B)** Retrieval, on day 1 and day 2, followed a remember/know recollection paradigm, see text. The frontal-view photographs of unfamiliar adult faces were used before (Bunzeck et al., 2006) and taken from “The Psychological Image Collection at Stirling (PICS, http://pics.psych.stir.ac.uk/)”.

The experiment took place on two consecutive days. On the first day, subjects participated in an encoding task and a subsequent recognition memory test, which was repeated on day two (with different stimuli, see below). During encoding, they were presented with 80 male and female faces (in random order) for 1 s, followed by a fixation cross for 2 s (Figure 1). Subjects were instructed to indicate *via* button presses as quickly and correctly as possible whether the face was male or female. One of both categories was associated with a reward, which was directly presented upon correct responses (1€). Incorrect responses and correct responses to the not-rewarded category led to no reward feedback (0€). The rewarded category was counterbalanced between participants. In a short training session, reward probability was 100% but it was 80% in the actual experiment. Participants were not explicitly instructed about which sex (male vs. female) was linked to a reward but quickly learned it throughout the training session. The encoding phase (including training) lasted approximately 20 min.

Shortly after encoding, participants took part in a first retrieval test. Here, 40 studied (i.e., old) faces from the encoding task were intermixed with 40 unstudied (i.e., new) faces. Each image was presented for 1.5 s and participants had up to 4 s to decide whether the face was “old” or “new.” Following a new response, they indicated whether they were “sure” or “guessed” in their decision. If they had recognized the face from before (i.e., old response), they had 4 s to decide if they “remembered,” “knew,” or just “guessed.” They chose “remember” when they recognized a picture and could recollect specific thoughts or associations linked to the study episode (recollection). They chose “know” when they recognized the picture but were not able to recall specific details or associations related to the study episode (familiarity). “Guessed” had to be pressed when they did not know whether a picture was old or new. Participants were carefully instructed, orally, and in writing, about the meaning of each response option.

On the second day, participants took part in a second recognition memory task, which was identical to the recognition memory test on day 1, except that it included the second, unseen, half of the studied items and 40 new unstudied distractors.

The frontal-view photographs of unfamiliar adult faces were used before Bunzeck et al. (2006) and taken from “The Psychological Image Collection at Stirling (PICS, http://pics.psych.stir.ac.uk/”).

### Genotyping

DNA samples were extracted by the Department of Human Genetics of the University Medical Center Hamburg-Eppendorf. Subjects were genotyped for the DAT1 40 bp, DAT1 30 bp, DRD4 48 bp, and CNR1 polymorphism. Genotyping was performed by Bioglobe (Hamburg, Germany). To detect the SNPs, the iPLEX^®^ method and the MALDI-TOF mass spectrometry were used on the MassARRAY^®^ system. A standard protocol, which was recommended by the system supplier, was used for most iPLEX reactions. It produces allele-specific analytes in a primer extension reaction applying a primer directly adjacent to the SNP site. The length of the extended primers is identical for any allele of interest so that detection and allelic discrimination rely on the present mass differences of each nucleotide base. For data acquisition, a MassARRAY^®^ Analyzer Compact was used before an automated data analysis with TYPER^®^ RT software version 3.4.

A polymerase chain reaction (PCR) amplification was used to analyze the different lengths of the VNTR. A fluorescent dye was attached to one of the primers and purification was performed. The obtained PCR product was separated by capillary electrophoresis on an ABI 3500 XL sequencing instrument. With the fluorescence signal, the length of the product was obtained and used to extrapolate the alleles from the raw data.

### Statistical Analysis

For the statistical analysis, IBM SPSS Statistics for Mac (Version 22.0) was used. Results were considered to be significant at *p* < 0.05 (in combination with Bonferroni correction for multiple comparisons when applicable, see “Results” section). As a measure of effect size, partial *η*^2^ is reported. Outliers were not excluded due to the large sample size. If the normal distribution, tested with the Shapiro–Wilk-test, was violated, nonparametric tests were used. The homogeneity of variances was examined with the Levene-test. When the sphericity assumption, measured with the Mauchly sphericity test, was violated, Greenhouse–Geisser correction was used.

For the encoding phase, mean RT was calculated for each participant for the rewarded and not-rewarded trials. The number of correct responses in the rewarded and non-rewarded trials was used as a measure of accuracy.

For the retrieval phase, corrected hit rates (CHR) were calculated for rewarded and not-rewarded stimuli separately, taking into account also the time of retrieval (day 1 vs. day 2) and type of memory (remember vs. know). This resulted in eight dependent variables per participant. CHR was calculated separately for remember responses (CHR-rem) and know responses (CHR-know) based on hit rates (percentage of correct old classifications) minus false alarm rate (i.e., false classification of old faces as new). Items classified as “guessed” were not included in any analysis. Genotypes were classified as described earlier and grouped based on previous studies.

The first set of analyses focused on RT, the number of correct responses, and memory performance independent of genotypes. Since RT and the number of correct answers for rewarded and not-rewarded trails were not normally distributed, the Wilcoxon-Test was used as a nonparametric test. For further analyses, a condition (rewarded/not-rewarded) × genotype ANOVA was performed to investigate whether genotype groups differed in RT and accuracy depending on the reward. In a final set of ANOVAs, memory performance was used as dependent variable. The factors included: genotype (between-subject variable), condition (rewarded/not-rewarded), time (day 1/day 2), and quality of memory (CHR-rem/CHR-know). To further investigate significant effects, Bonferroni corrected *post hoc t*-tests were performed.

Genotype groups were also compared regarding socio-demographic variables. For metric variables, an ANOVA was used and for alternative and nominal data the *χ*^2^-test.

### Other Polymorphisms

Three other polymorphisms were analyzed *post hoc* in an exploratory fashion. However, they were not planned to be part of this manuscript and will, therefore, not be reported in detail here: dopamine receptor D4 polymorphism 120 bp, noradrenergic receptor polymorphism α2B, and serotonin transporter-linked polymorphic region: 5-HTTLPR.

## Results

### Genotyping and Group Descriptions

Genotyping was successful in most participants, group sizes range from 646 to 662. Distributions for the different polymorphism are shown in Table 1. Groups did not differ in age (*p*’s > 0.058), gender (*p*’s > 0.061), BMI (*p*’s > 0.582), smoking (*p*’s > 0.210), alcohol consumption regarding glasses/week (*p*’s > 0.083; DRD4 48 bp: *p* = 0.038) and years of consumption (*p*’s > 0.085) and cannabis consumption (*p*’s > 0.137), see Table 1. Note that the *p*-values reported here and in Table 1 are uncorrected *p*-values.

**Table 1:** Socio demographic variables/polymorphism groups.

Polymorphism	Group size (%)	Age (*M, SD*)	Gender female (%)	BMI (*M, SD*)	Smoking (%)	Alcohol consump.		Cannabis consump. (%)
						(glasses/week)	years	
**DAT1 40 bp**	*n* = 656	*p* = 0.058	*p* = 0.281	*p* = 0.582	*p* = 0.615	*p* = 0.835	*p* = 0.085	*p* = 0.137
Homozygous 9-R allele	44 (6.5)	25.5 (3.17)	30 (68.2)	*n* = 44	*n* =44	*n* = 38		*n* = 42
				23.20 (3.11)	11 (25.0)	3.53 (2.94)	9.47 (3.46)	11 (26.2)
Heterozygous	239 (35.7)	24.18 (3.23)	179 (74.9)	*n* = 228	*n* = 237	*n* = 208		*n* = 236
				22.67 (3.24)	49 (20.7)	3.63 (3.07)	8.08 (3.19)	33 (14.0)
Homozygous10-R allele	373 (56.9)	24.4 (3.49)	258 (69.2)	*n* = 351	*n* = 367	*n* = 320		*n* = 368
				22.67 (3.32)	70 (19.1)	3.47 (3.14)	8.51 (3.79)	59 (16.0)
**DAT1 30 bp**	*n* = 662	*p* = 0.163	*p* = 0.061	*p* = 0.680	*p* = 0.472	*p* = 0.083	*p* = 0.251	*p* = 0.818
Homozygous 4-R allele	31 (4.7)	24.39 (3.17)	22 (71.0)	*n* = 30	*n* = 31	*n* = 26		*n* = 31
				22.79 (3.26)	5 (16.1)	2.62 (2.25)	8.33 (3.74)	6 (19.4)
Heterozygous	219 (33.1)	24.07(3.35)	169 (77.2)	*n* = 211	*n* = 217	*n* = 194		*n =* 214
				22.54 (3.09)	50 (23.0)	3.86 (3.18)	8.04 (3.52)	33 (15.3)
Homozygous 5–R allele	412 (62.2)	24.52 (3.42)	281 (68.2)	*n* = 388	*n* = 406	*n* = 368		*n* = 407
				22.78 (3.37)	79 (19.4)	3.42 (3.07)	8.59 (3.56)	65 (16.0)
**DRD4 48 bp**	*n* = 646	*p* = 0.244	*p* = 0.244	*p* = 0.734	*p* = 0.917	*p* = 0.832	*p* = 0.038	*p* = 0.649
Short (7-R allele absent)	434 (67.2)	24.30 (3.28)	311 (71.7)	*n* = 412	*n* =430	*n* = 376		*n* = 426
				22.68 (3.39)	88 (20.5)	3.36 (2.99)	8.45 (3.54)	72 (16.9)
Long (7-R allele present)	212 (32.8)	24.63 (3.60)	152 (71.7)	*n* = 201	*n* = 208	*n* = 183		*n* = 210
				22.77 (3.05)	44 (21.2)	3.93 (3.32)	8.35 (3.53)	32 (15.2)
**CNR1**	*n* = 661	*p* = 0. 807	*p* = 0.645	*p* = 0.791	*p* = 0.210	*p* = 0. 905	*p* = 0. 231	*p* = 0.988
Homozygous short (≤12-R/≤12-R)	70 (10.6)	24.21 (3.18)	53 (75.7)	*n* = 65	*n* = 68	*n* = 60		*n* = 68
				22.62 (3.18)	18 (26.5)	4.18 (3.02)	8.19 (2.79)	11 (16.2)
Heterozygous (≤12-R/>12)	286 (43.3)	24.45 (3.43)	205 (71.7)	*n* = 273	*n* = 282	*n* = 247		*n* = 281
				22.80 (3.19)	50 (17.7)	3.44 (3.01)	8.42 (3.71)	46 (16.4*)*
Homozygous long (>12-R/>12-R)	305 (46.1)	24.31 (3.40)	214 (70.2)	*n* = 290	*n* = 303	*n* = 264		*n* = 302
				22.62 (3.38)	66 (21.8)	3.49 (3.16)	8.41 (3.56)	48 (15.0)

*Note. BMI, body mass index; bp, base pair; CNR1, cannabinoid receptor type 1; DAT1, DA transporter polymorphism; DRD4, DA receptor type 4; l, long; s, short; *n*, group size; * M*, mean; *SD*, standard deviation*. *p*-values are uncorrected for multiple comparisons.

Apart from the numbers reported in Table 1, we also investigated the relative distribution of the different alleles and tested for the Hardy–Weinberg equilibrium (HWE; Graffelman and Weir, 2018). It revealed for DAT1 40 bp: 10-R 75.4%, 9-R 24.6%, HWE *χ*^2^ = 0.079, *p* = 0.78; for DAT 30 bp: 5-R 78.8%, 4-R 21.2%, HWE *χ*^2^ = 0.012, *p* = 0.91; DRD4 48 bp: 7 R 18%, < 7 R 82%, HWE *χ*^2^ = 0.0007, *p* = 0.97; and CNR1 bp: >12-R 67.8%, ≤ 12-R 32.2%, HWE *χ*^2^ = 0.008, *p* = 0.93. Taken together, the observed genotypic distribution of our SNPs did not significantly deviate from expectation according to the Hardy–Weinberg equilibrium (*p* > 0.05).

### Behavioral Data

#### Reaction Time and Accuracy

**Figure 2:**
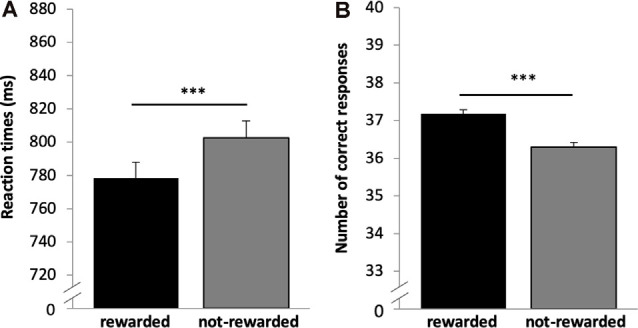
Results for the reward task (encoding). **(A)** Reward dependent effect on reaction times. Participants responded faster for rewarded as compared to not-rewarded trials. **(B)** The number of correct responses for rewarded and not-rewarded trials. Participants were more accurate in the rewarded trials. ***p < 0.001, *n* = 669.

Reaction time and accuracy analysis included *n* = 669 subjects. Both variables were not normally distributed (*p* < 0.001); therefore, the non-parametric Wilcoxon-test was used. As expected, participants responded significantly faster in rewarded (778.42 ms, SD = 249.89) as compared to not-rewarded trials [804.61 ms, SD = 259.45, *p* < 0.001, *r* (Wilcoxon test effect size) = 0.312], see Figure 2A; and they were more accurate in the rewarded 37.19 (2.69) as compared to the not-rewarded 36.30 (3.19) trials (*p* < 0.001, *r* = 0.227, Figure 2B).

#### Memory

A 2 × 2 × 2 repeated-measures ANOVA (*n* = 669) on CHR as dependent variable and the within subject factors reward (rewarded vs. not-rewarded), quality of memory (recollection vs. familiarity) and time (day 1 vs. day 2) showed significant main effects of time (*F*_(1,668)_ = 115.136, *p* < 0.001, *η*^2^ = 0.147) and quality of memory (*F*_(1,668)_ = 42.588, *p* < 0.001, *η*^2^ = 0.060). However, there was no main effect of reward (*F*_(1,668)_ = 1.604, *p* < 0.206). The interaction between time × quality of memory was also significant (*F*_(1,668)_ = 17.346, *p* < 0.001, *η*^2^ = 0.025), while all other interactions were not (*p* > 0.05).

**Figure 3:**
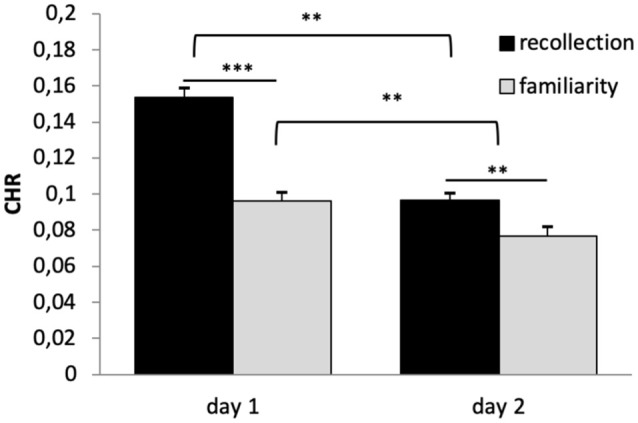
Recognition memory performance. Recognition memory decreased from day 1 to day 2. Corrected hit rates (CHR)-rem were higher as compared to CHR-know, and this difference was more pronounced on day 1. **p < 0.01, ***p < 0.001.

Bonferroni corrected *post hoc* analyses (pairwise comparisons) revealed a significant difference between recollection and familiarity scores (CHR) on both days (*p*’s < 0.006), and that memory performance (both categories) differed significantly between days (*p*’s < 0.003), see Figure 3. Specifically, memory was lower on day 2 compared with day 1, and CHR-rem were higher as compared to CHR-know. The interaction between time × quality of memory was driven by a larger difference between CHR-rem vs. CHR-know on day 1 as compared to day 2.

#### DA Transporter 40 bp Polymorphism

In the following analysis, *n* = 656 participants were included. Subjects were subdivided into three groups: homozygous 9-R allele, heterozygous and homozygous 10-R allele. For information about socio-demographic variables, see Table 1.

##### Reaction Time and Accuracy

The 2 × 3 ANOVA with the factors reward and genotype on RT during encoding revealed a significant main effect of reward (*F*_(1,653)_ = 27.522, *p* < 0.001, *η*^2^ = 0.040) but no main effect of genotype (*F*_(2,653)_ = 0.831, *p* = 0.436) and no significant interaction (*F*_(2,653)_ = 0.740, *p* = 0.478). When accuracy was used as a dependent variable, there was a main effect of reward (*F*_(1,653)_ = 15.377, *p* < 0.001, *η*^2^ = 0.023) but no main effect of genotype (*F*_(1,653)_ = 0.058, *p* = 0.943) and no significant interaction (*F*_(2,653)_ = 0.061, *p* = 0.941).

#### Recognition Memory

**Figure 4:**
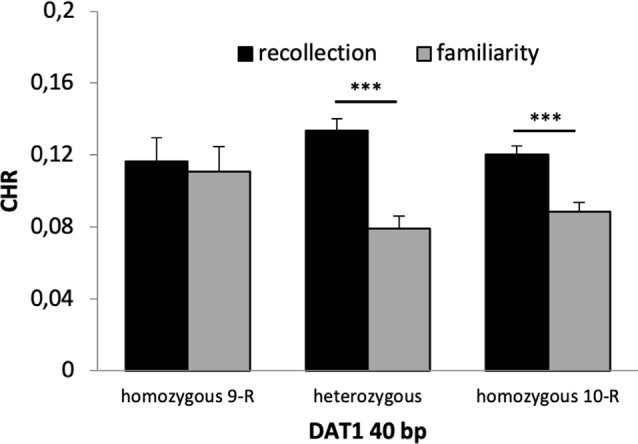
Recognition memory performance for the DAT1 40 bp genotype. Heterozygous and homozygous 10-R, but not homozygous 9-R (*p* = 0.803), showed significantly higher CHR-rem vs. CHR-fam (*p*’s < 0.001). ***p < 0.001.

A 2 × 2 × 2 × 3 ANOVA with the factors time, reward, quality of memory and genotype revealed a significant main effect of time (*F*_(1,653)_ = 56.130, *p* < 0.001, *η*^2^ = 0.079) and quality of memory (*F*_(1,653)_ = 12.190, *p* = 0.001, *η*^2^ = 0.018) but no main effect of reward (*F*_(1,653)_ = 0.441 *p* = 0.507) or genotype (*F*_(2,653)_ = 0.491, *p* = 0.608). There was a significant interaction between time × quality of memory (*F*_(2,653)_ = 14.324, *p* < 0.001, *η*^2^ = 0.021) and a marginally significant interaction between quality of memory × genotype (*F*_(1,653)_ = 2.673, *p* = 0.070, *η*^2^ = 0.008). *Post hoc t*-tests revealed that this latter interaction was driven by significant differences between CHR-rem and CHR-know in heterozygous and 10-R homozygous (*p*’s < 0.001) but not in the homozygous 9-R (*p* = 0.803, see Figure 4). All other interactions were not statistically significant (*p* > 0.1).

#### DA Transporter 30 bp Polymorphism

In the following analysis, *n* = 662 participants were included. Subjects were subdivided into three groups: 4-R homozygotes, heterozygotes, and 5-R homozygotes. For information about socio-demographic variables, see Table 1.

##### Reaction Time and Accuracy

**Figure 5:**
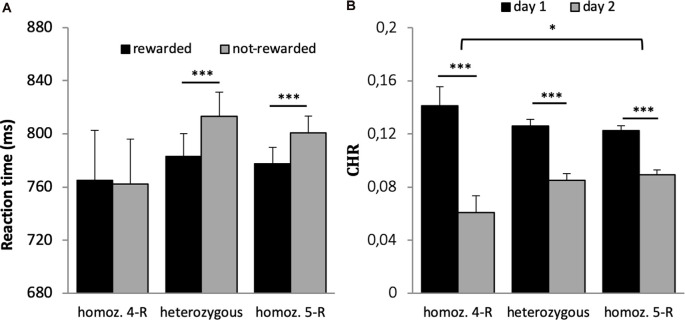
**(A)** Reaction time of the DAT1 30 bp genotype. A significant interaction between group and reward was driven by faster responses for rewarded vs. not-rewarded items in the heterozygous and homozygous 5-R group (*p* < 0.001) but not in the homozygous 4-R group (*p* = 0.882). **(B)** Differences in memory performance of the DAT1 30 bp genotype. Recognition memory performance decreased over time. However, this difference was most pronounced in the homozygous 4-R group. *p < 0.05; ***p < 0.001.

The 2 × 3 ANOVA with the factors reward and genotype on reaction time revealed a significant effect of reward (*F*_(1,659)_ = 58.406, *p* < 0.001, *η*^2^ = 0.019), a significant reward × genotype interaction (*F*_(1,659)_ = 3.083, *p* = 0.046, *η*^2^ = 0.009) but no main effect of genotype (*F*_(1,659)_ = 0.282, *p* = 0.754). *Post hoc t*-tests revealed that the interaction was driven by significantly faster response times for rewarded vs. not-rewarded trials in the heterozygous and homozygous 5-R group (*p* < 0.001) but not in the homozygous 4-R group (*p* = 0.882, see Figure 5A). When accuracy was used as dependent variable in the 2 × 3 ANOVA, there was a main effect of reward (*F*_(1,659)_ = 6.400, *p* = 0.012, *η*^2^ = 0.010) but no main effect of genotype (*F*_(2,659)_ = 0.299, *p* = 0.742) and no interaction with genotype (*F*_(1,659)_ = 1.482, *p* = 0.228).

##### Recognition Memory

A 2 × 2 × 2 × 3 ANOVA with the factors time, reward, quality of memory and genotype revealed a significant main effect of time (*F*_(1,659)_ = 72.817, *p* < 0.001, *η*^2^ = 0.100) and quality of memory (*F*_(1,659)_ = 5.562, *p* = 0.019, *η*^2^ = 0.008), but no main effect of reward (*F*_(1,659)_ = 2.143, *p* = 0.144) or genotype (*F*_(2,659)_ = 0.097, *p* = 0.908). There was a significant interaction between time × quality of memory (*F*_(1,659)_ = 9.783, *p* = 0.002, *η*^2^ = 0.015) and time and genotype (*F*_(2,659)_ = 4.051, *p* = 0.018, *η*^2^ = 0.012).

All three groups had significantly higher memory scores on day 1 (*p*’s < 0.001) as compared to day 2. The differences between both retrieval days were significantly larger in the homozygous 4-R group compared to the homozygous 5-R group (see Figure 5B; Bonferroni corrected *post hoc t*-tests). No other interactions were statistically significant (*p* > 0.9).

#### DA Receptor D4 48 bp Polymorphism

In the following analysis, *n* = 646 participants were included. We subdivided the sample into two groups based on the 7-R allele: participants carrying at least one 7-R allele were categorized as “long” while all others were categorized as “short” group (Asghari et al., 1995; Eisenberg et al., 2007; Dreber et al., 2009). For information about socio-demographic variables, see Table 1.

##### Reaction Time and Accuracy

The 2 × 2 ANOVA with the factors reward and genotype on reaction time revealed a significant effect of reward (*F*_(1,651)_ = 58.406, *p* < 0.001, *η*^2^ = 0.082) but no effect of genotype (*F*_(1,651)_ = 1.847, *p* = 0.175) and no interaction (*F*_(1,651)_ = 1.098, *p* = 0.295). When accuracy was used as a dependent variable in the 2 × 2 ANOVA there was a main effect of reward (*F*_(1,651)_ = 28.588, *p* < 0.001, *η*^2^ = 0.042) but no effect of genotype (*F*_(1,651)_ = 0.399, *p* = 0.528) and no interaction (*F*_(1,651)_ = 0.826, *p* = 0.364).

##### Recognition Memory

**Figure 6:**
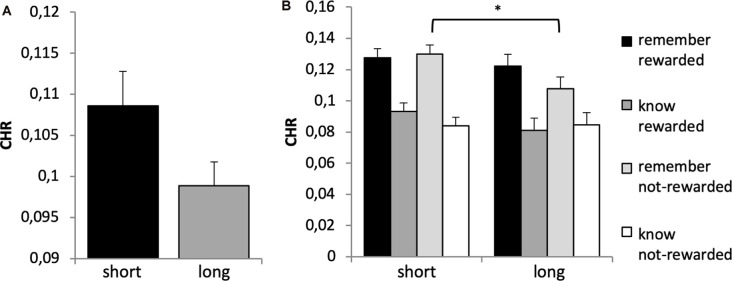
**(A)** Memory performance for DRD4 48 bp polymorphism. Based on the main effect of group (see text), a *post hoc t*-test revealed a trend for higher CHR for the short allele carriers (7-repeat allele absent) as compared to the long allele carriers (*t*_ 644_ = −1.928, *p* = 0.054). **(B)** Reward × quality of memory × genotype interaction. Bonferroni corrected *post hoc* analyses revealed significantly lower CHR-rem in the long as compared to the short allele group for not-rewarded items only (*p* = 0.026). There were no differences between groups in all other categories (*p*’s > 0.209). Short refers to 7-allele absent, and long to 7-allele present in the DRD4 48 bp, *p < 0.05.

A 2 × 2 × 2 × 2 ANOVA with the factors time, reward, quality of memory and genotype revealed a significant main effect of time (*F*_(1,651)_ = 90.603, *p* < 0.001, *η*^2^ = 0.122), quality of memory (*F*_(1,651)_ = 31.861, *p* < 0.001, *η*^2^ = 0.047) and a borderline significant effect for the between-subject factor genotype (*F*_(1,651)_ = 3.795, *p* = 0.052, *η*^2^ = 0.006), see Figure 6A. This was driven by a trend for significantly higher CHR (collapsed across remember and know responses) for the short vs. long group (*t*-test: *p* = 0.054; Bonferroni corrected). There was no main effect of reward (*F*_(1,651)_ = 1.351, *p* = 0.246).

There was a significant time × quality of memory interaction (*F*_(2,651)_ = 14.591, *p* < 0.001, *η*^2^ = 0.022) and a significant reward × quality of memory × genotype interaction (*F*_(1,651)_ = 3.868, *p* = 0.050, *η*^2^ = 0.008). This latter interaction was further explored with *post hoc* tests, which indicated a difference between genotype groups regarding non rewarded recollection-based memory performance, see Figure 6B. No other interactions were statistically significant (*p* > 0.3).

#### Cannabinoid Receptor Type 1

In the following analysis, *n* = 661 participants were included. Subjects were subdivided into three groups (Comings, 1998) based on the 12-repeat allele (Ruiz-Contreras et al., 2013). More than 12-repeat alleles were categorized as long, resulting in three different groups: homozygous short (≤12-R/≤12-R), heterozygous (≤12-R/>12-R), and homozygous long (>12-R/>12-R). For more information about socio-demographic variables, see Table 1.

##### Reaction Time and Accuracy

The 2 × 3 ANOVA with the factors reward and genotype on reaction time revealed a significant effect of reward (*F*_(1,658)_ = 50.930, *p* < 0.001, *η*^2^ = 0.072) but no main effect of genotype (*F*_(2,658)_ = 0.566, *p* = 0.568) and no significant interaction (*F*_(2,658)_ = 0.156, *p* = 0.856). When accuracy was used as dependent variable in the 2 × 3 ANOVA, there was a main effect of reward (*F*_(1,658)_ = 28.933, *p* < 0.001, *η*^2^ = 0.042) but no main effect of genotype (*F*_(2,658)_ = 0.927, *p* = 0.396) and no significant interaction (*F*_(2,658)_ = 0.584, *p* = 0.579).

##### Recognition Memory

**Figure 7:**
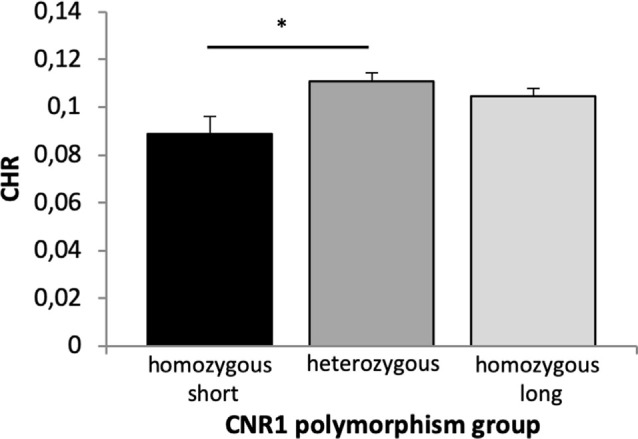
Memory performance in the CNR1 polymorphism. Based on a main effect of group (see text), Bonferroni corrected *post hoc t*-tests revealed significant differences in CHR between homozygous short and heterozygous group (*p* = 0.018) but not between homozygous short and long (*p* = 0.144) and homozygous long and heterozygous group (*p* = 0.615) for overall memory performance. CHR, corrected hit rates; CNR1, cannabinoid receptor type 1, *p < 0.05.

A 2 × 2 × 2 × 3 ANOVA with the factors time, reward, quality of memory and genotype revealed a significant main effect of time (*F*_(1,658)_ = 62.715, *p* < 0.001, *η*^2^ = 0.087), quality of memory (*F*_(1,658)_ = 20.895, *p* < 0.001, *η*^2^ = 0.031) and genotype (*F*_(2,658)_ = 3.872, *p* = 0.021, *η*^2^ = 0.012). Bonferroni corrected *post hoc* tests revealed a significantly lower CHR in the homozygous short as compared to the heterozygous group (*p* = 0.018, Figure 7).

As in all previous analyses, there was a significant interaction between time × quality of memory (*F*_(1,658)_ = 6.684, *p* = 0.010, *η*^2^ = 0.010) but no significant main effect of reward. All other interactions were not statistically significant (*p* > 0.16).

## Discussion

In a reward-based long-term recognition memory task and a sample of 669 healthy human adults, we can show that reward accelerates response times and accuracy. While only one genotype polymorphism, namely DAT 30 bp, could be linked to reward dependent response times during encoding, reward had no impact on declarative long-term recognition memory and there was no interaction with any of the genotypes tested here. However, there were differential effects of genotype polymorphisms on declarative long-term memory: (a) DAT1 40 bp was linked to the quality of memory with a more pronounced difference between recollection and familiarity in the heterozygous and homozygous 10R as compared to homozygous 9-R; (b) DAT1 30 bp was linked to memory decay, which was most pronounced in homozygous 4-R; (c) DRD4 48 bp was linked to overall recognition memory with higher performance in the short allele group; and (d) CNR1 was linked to overall memory with reduced performance in the homozygous short group. These findings give new insights into how polymorphisms, that are related to dopaminergic neuromodulation, affect different aspects of long-term recognition memory performance.

As expected, cues that predict monetary rewards invigorate and drive behavioral performance (Figure 2). Specifically, this was expressed in faster response times and higher accuracy during encoding, which is in accordance with previous results (Knutson et al., 2001; Pessiglione et al., 2007; Bayer et al., 2013; Steiger and Bunzeck, 2017). At the physiological level, these effects may be linked to the dopaminergic system as suggested by computational models and empirical studies in animals and human subjects (Niv et al., 2007; Guitart-Masip et al., 2011; Dayan, 2012; Beierholm et al., 2013). For instance, individual reward-related response times in older participants could be predicted by the structural integrity of the dopaminergic substantia nigra/ventral tegmental area (SN/VTA) as measure by magnetization transfer imaging (Steiger and Bunzeck, 2017). In line with a DA-reward hypothesis (Wise, 1982), here we found a link between response times and DAT 30 bp. The heterozygous and homozygous 5-R groups showed significantly faster response times for rewarded trials as compared to not-rewarded trials; however, no such effect was observed in the homozygous 4-R group (Figure 5A). This may suggest impaired neural responses to reward predicting cues in the homozygous 4-R group, which could be further investigated with functional brain imaging such as fMRI or PET.

In contrast to our predictions, there was no effect of reward on recognition memory, and no interaction with genotype. The former hypothesis was based on the assumption that reward not only drives response times and physical effort but also cognitive performance including long-term memory. For instance, retrieval performance was increased for rewarded scenes images (Wittmann et al., 2005; Adcock et al., 2006), words (Gruber and Otten, 2010), or photographs (Shigemune et al., 2010), and everyday objects with different motivational value (Schomaker and Wittmann, 2017). However, not all studies showed a positive effect of reward on cognition, including learning and memory (Callan and Schweighofer, 2008; Ariely et al., 2009; Sharifian et al., 2017; Steiger and Bunzeck, 2017), and some have even reported detrimental effects (Mobbs et al., 2009; Chib et al., 2012; Kuhbandner et al., 2016). While psychological explanations, for instance, related to over motivation or anxiety, might help to explain these opposing results (Callan and Schweighofer, 2008; Mobbs et al., 2009), they might also relate to differences in task design. Specifically, tasks with cues that initially indicate a reward for correct subsequent retrieval (Adcock et al., 2006; Gruber and Otten, 2010; Wittmann et al., 2013) appear to be more robust as compared to designs used here, in which a cue predicts an immediate reward for a correct response (see also Steiger and Bunzeck, 2017).

Along the same lines, although the effect of reward on memory has been shown for several stimulus materials (see above) and retention intervals—including 24 h (Krebs et al., 2009; Bunzeck et al., 2012), it remains unclear whether the faces used here might be special. In particular, we presented male and female faces without scalp hair and background information, which typically helps to form associations and therefore drives recollection. Moreover, the reward task was administered in the context of several other tasks (some of which also included reward) over 2 days (see “Materials and Methods” section), which might lead to interferences and therefore reduced the effects of reward on long-term recognition memory. Indeed, on average the CHRs were rather low (Figure 3). In any case, a parsimonious explanation for our absent interaction between reward-based long-term memory and genotype might relate to the overall absence of a reward effect on long-term memory. Therefore, further studies should include a task with a more robust reward effect.

The main finding of our study is that dopaminergic genotypes had differential effects on several aspects of declarative long-term recognition memory. First, there was a significant main effect of memory with higher recollection as compared to familiarity scores, and, importantly, this effect interacted with the DAT 40 bp genotype. Specifically, recollection was enhanced in heterozygous participants and carriers of the homozygous 10-R; however, there was no significant difference between recollection and familiarity scores in homozygous 9-R carriers (Figure 4). This observation is consistent with dual-process models of recognition memory suggesting a distinction between recollection and familiarity (Yonelinas et al., 2010). While the hippocampus and posterior parahippocampal gyrus are closely associated with recollection, the anterior parahippocampal gyrus is more associated with familiarity (Ranganath et al., 2004; Bowles et al., 2007; Diana et al., 2007; Sauvage et al., 2008; Vann et al., 2009; Martin et al., 2013). Therefore, the hippocampus appears to be more critical for recollection as compared to familiarity. Concerning genetic variations, the DAT 9-R allele has been associated with stronger SN/VTA activity in an episodic memory task, which was, however, not present at the behavioral level (Schott et al., 2006). In contrast, another study could link the DAT 10-R allele to hippocampal activity (Bertolino et al., 2008), which is more compatible with our findings of enhanced recollection in the homozygous 10-R but not the homozygous 9-R group. Therefore, our findings support dual-process models and they suggest that variations in the DAT1 40 bp polymorphisms contribute to interindividual differences in recollection- and familiarity-based recognition memory possibly *via* hippocampal activity and dopaminergic neurotransmission.

Second, recognition memory significantly decreased from day 1 to day 2, which is in line with the notion of a memory decay over time (e.g., Schandry, 2011), and, importantly, this effect interacted with the DAT1 30 bp polymorphisms (Figure 5B). The decay of recognition memory over 2 days was most pronounced in the homozygous 4-R group, or, conversely, less pronounced in the homozygous 5-R group. Past research has associated the DAT1 30 bp polymorphism 5-R allele with impulsivity (Paloyelis et al., 2010), ADHD (Asherson et al., 2007; Simpson et al., 2010), and addiction (Guindalini et al., 2006; O’Gara et al., 2007; Smirnova et al., 2011), possibly through the modulation of dopaminergic processes. The present findings suggest that the 4-R allele affects recognition memory by enhanced forgetting rates. At the physiological level, this might be related to higher expression of the DAT in 4-R carriers, leading to decreased DA in the synaptic cleft and, therefore, less neurotransmission. Conversely, homozygous 5-R carries may express more DAT, leading to temporally more stable memory representations.

Third, overall recognition memory performance was higher in short allele carriers of the DRD4 48 bp polymorphism as compared to long allele carriers (at least one 7-R allele, Figure 6). This is partly consistent with previous research demonstrating decreased working memory performance in children carrying the 7-R allele (Froehlich et al., 2007; Altink et al., 2012). In contrast, others found better performance in cognitive tasks, including short-term working memory, in 7-R allele carriers (Boonstra et al., 2008), which might be mediated by ADHD status and age (Altink et al., 2012). Alternatively, the DRD4 gene might be relevant for attention selection of highly relevant information. This has been suggested since long allele carriers demonstrated increased selective attention to “high-priority items” in a category learning and operation span task (Gorlick et al., 2015). A similar attention effect with advantages for long allele carriers has been shown in the context of emotional faces (Wells et al., 2013). Both studies (Wells et al., 2013; Gorlick et al., 2015) would predict a clear behavioral advantage for rewarded (i.e., high priority) items in the long allele group. However, this was not the case here. Together, in our study, the short allele carriers of the DRD4 48 bp polymorphism showed overall enhanced recognition memory performance, which fits the notion that DA plays a role in encoding novel information into long-term memory (Lisman and Grace, 2005; Lisman et al., 2011). However, this size of the effect—like most others we observed (see Table 1) was rather weak and needs to be replicated in future studies.

Fourth, variations in the CNR1 polymorphism were linked to overall memory with reduced performance in the homozygous short group (Figure 7A). More specifically, carriers of the short homozygous allele (≤12-R/≤12-R) showed decreased memory performance compared to the heterozygous group (≤12-R/>12). This effect contrasts a previous study showing increased performance in working memory in the short homozygous group (Ruiz-Contreras et al., 2013), but it supports the more general observation that the CNR1 polymorphism modulates memory performance (Ruiz-Contreras et al., 2011). At the physiological level, activation of CNR1 receptors in the ventral hippocampus of rats enhanced neuronal firing of DA neurons in the VTA and, at the same time, decreased activity of non-DA neurons; further, it increased reward salience and impaired social behaviors (Loureiro et al., 2015). Since the CNR1 polymorphism has also been associated with reward-related traits and disorders (Ponce et al., 2003; Martínez-Gras et al., 2006; Chavarría-Siles et al., 2008), we expected an interaction with reward processing. However, those studies have been inconsistent in their association with a specific allele. For instance, the allelic distribution of the CNR1 seems to be heterogeneous among populations (Ruiz-Contreras et al., 2011), and alleles that were associated with reward-related behavior were not even present in this sample so that their influence could not be detected. Taken together, in line with the notion of a link between CNR1 and the dopaminergic system, our findings suggest that CNR1 modulates long-term memory processes possibly through modulation of DA firing.

We would like to point out that several studies we mentioned above to interpret our findings do not necessarily relate to declarative long-term memory but instead to working memory (or other cognitive domains such as attention). According to traditional models, declarative long-term and working memory rely on partly overlapping but—importantly—distinct brain regions (Squire et al., 2004; Lara and Wallis, 2015; Squire and Dede, 2015; Miller et al., 2018). Specifically, while studies on working memory emphasize the role of the prefrontal cortex, models of declarative long-term memory focus on the medial temporal lobe. Under the assumption that genes and associated polymorphisms may also act in a regionally specific manner (e.g., Schott et al., 2006; Yacubian et al., 2007), the parallels we draw need to be treated with caution. To investigate this issue further, future studies are required.

The effects of DA are not only limited to novelty encoding, but it also modulates long-term memory *via* consolidation and retrieval activity. For instance, in Wittmann et al. (2005), the behavioral effects of reward on recognition memory were most pronounced after a retention interval of 3 weeks, which is consistent with a role of DA in the late phase of long-term potentiation (LTP, see e.g., Lisman and Grace, 2005). Along the same lines, research in animals could show that reward enhances hippocampal reactivation (Singer and Frank, 2009) and hippocampal DA regulates the persistence of long-term memory (Rossato et al., 2009). Finally, pharmacological modulation of the dopaminergic system in humans drives memory retrieval, which provides evidence for a role of DA in episodic memory retrieval (Clos et al., 2019a,b). Concerning our findings, this suggests that polymorphisms affecting dopaminergic neuromodulation may relate to encoding, consolidation, and/or retrieval. A clear distinction between all three components was not possible here and should, therefore, be addressed in future studies for instance with fMRI.

We acknowledge that several genotypes, other than the ones investigated here, also relate to dopaminergic neuromodulation and as such could impact learning and memory processes. These include, for instance, COMT Val158Met (Bilder et al., 2004); DRD2 (Richter et al., 2017), DRD3 (Papenberg et al., 2013), CHRNA4 (Markett et al., 2009), and DARPP-32 (Schuck et al., 2013; Persson et al., 2017). However, further investigating all potentially relevant genes is beyond the scope of the current study and would need to involve other methodological and statistical approaches.

Another important question that arises based on our results is the link and possible interaction between the investigated genes. Indeed, in a previous study, reward-related hemodynamic activity in the ventral striatum could be related to the combination of DAT and COMT polymorphisms (Yacubian et al., 2007). More recent work has confirmed such epistatic gene-gene interactions, for instance, by showing that COMT and DRD3 together modulate behavior in children with ADHD (Fageera et al., 2020) and that DRD4 in combination with COMT modulate the clinical responses to clozapine in schizophrenia patients (Rajagopal et al., 2018). In our study, the most apparent link to the investigated polymorphisms is their impact on dopaminergic neuromodulation. However, the exact processes, including the relationship to different types of memory, underlying brain regions, and possible gene-gene interactions, remain unclear and need to be addressed in future studies.

Finally, although the observed genotypic distribution of our SNPs did not significantly deviate from expectation according to the Hardy–Weinberg equilibrium, it should be noted that some effects appear to be driven by the less frequent homozygotes in three of the four variants investigated (DAT1 40 bp, DAT1 30 bp, CNR1), see Figures 4, 5, 7. Therefore, future work should replicate our findings.

Together, in a cohort of 669 healthy human adults, we can show that reward accelerates response times and accuracy, but it did not affect subsequent recognition memory. Four *a priori* selected genotypes, previously associated with the dopaminergic system, could be related to different aspects of recognition memory. Quality of memory was linked to DAT1 40 bp, memory decay was linked to DAT1, and overall recognition memory was linked to DRD4 48 bp and CNR1. As such, our findings give new insights into how interindividual differences in learning and memory processes relate to genes that modulate the activity of the dopaminergic system.

## Data Availability Statement

This manuscript contains previously unpublished data. The datasets generated for this study are available on request to the corresponding author.

## Ethics Statement

The studies involving human participants were reviewed and approved by the ethics committee of the Hamburg Medical Association. The participants provided their written informed consent to participate in this study.

## Author Contributions

CL and NB analyzed the data and wrote the manuscript. DS acquired the data. RK, TS, and NB designed the experiment. DS, RK, and TS proofread the manuscript. All authors contributed to the article and approved the submitted version.

## Conflict of Interest

RK receives advisory honoraria from JoyVentures, Herzlia, Israel.

The remaining authors declare that the research was conducted in the absence of any commercial or financial relationships that could be construed as a potential conflict of interest.

The handling editor declared a shared affiliation, though no other collaboration, with several of the authors DS, RK, TS, NB at time of review.

## References

[B1] Adcock R. A. Thangavel A. Whitfield-Gabrieli S. Knutson B. Gabrieli J. D. (2006). Reward-motivated learning: mesolimbic activation precedes memory formation. Neuron 50, 507–517. 10.1016/j.neuron.2006.03.036 16675403

[B2] Altink M. E. Rommelse N. N. J. Slaats-Willemse D. I. E. Väsquez A. A. Franke B. Buschgens C. J. M.. (2012). The dopamine receptor D4 7-repeat allele influences neurocognitive functioning, but this effect is moderated by age and ADHD status: an exploratory study. World J. Biol. Psychiatry 13, 293–305. 10.3109/15622975.2011.595822 22111665

[B3] Ariely D. Gneezy U. Loewenstein G. Mazar N. (2009). Large stakes and big mistakes. Rev. Econ. Stud. 76, 451–469. 10.1111/j.1467-937x.2009.00534.x

[B4] Asghari V. Sanyal S. Buchwaldt S. Paterson A. Jovanovic V. Van Tol H. H. (1995). Modulation of intracellular cyclic AMP levels by different human dopamine D4 receptor variants. J. Neurochem. 65, 1157–1165. 10.1046/j.1471-4159.1995.65031157.x 7643093

[B5] Asherson P. Brookes K. Franke B. Chen W. Gill M. Ebstein R. P.. (2007). Confirmation that a specific haplotype of the dopamine transporter gene is associated with combined-type ADHD. Am. J. Psychiatry 164, 674–677. 10.1176/ajp.2007.164.4.674 17403983

[B6] Ballon N. Leroy S. Roy C. Bourdel M. C. Charles-Nicolas A. Krebs M. O.. (2006). (AAT)*n* repeat in the cannabinoid receptor gene (CNR1): association with cocaine addiction in an African-Caribbean population. Pharmacogenomics J. 6, 126–130. 10.1038/sj.tpj.6500352 16314880

[B7] Bayer J. Bandurski P. Sommer T. (2013). Differential modulation of activity related to the anticipation of monetary gains and losses across the menstrual cycle. Eur. J. Neurosci. 38, 3519–3526. 10.1111/ejn.12347 23981052

[B8] Beierholm U. Guitart-Masip M. Economides M. Chowdhury R. Düzel E. Dolan R.. (2013). Dopamine modulates reward-related vigor. Neuropsychopharmacology 38, 1495–1503. 10.1038/npp.2013.48 23419875 PMC3682144

[B9] Benyamina A. Kebir O. Blecha L. Reynaud M. Krebs M. (2011). CNR1gene polymorphisms in addictive disorders: a systematic review and a meta-analysis. Addict. Biol. 16, 1–6. 10.1111/j.1369-1600.2009.00198.x 20192949

[B10] Bertolino A. Di Giorgio A. Blasi G. Sambataro F. Caforio G. Sinibaldi L.. (2008). Epistasis between dopamine regulating genes identifies a nonlinear response of the human hippocampus during memory tasks. Biol. Psychiatry 64, 226–234. 10.1016/j.biopsych.2008.02.001 18374902

[B11] Bilder R. M. Volavka J. Lachman H. M. Grace A. A. (2004). The catechol-O-methyltransferase polymorphism: relations to the tonic-phasic dopamine hypothesis and neuropsychiatric phenotypes. Neuropsychopharmacology 29, 1943–1961. 10.1038/sj.npp.1300542 15305167

[B12] Boonstra A. M. Kooij J. J. S. Buitelaar J. K. Oosterlaan J. Sergeant J. A. Heister J. G. A. M. A.. (2008). An exploratory study of the relationship between four candidate genes and neurocognitive performance in adult ADHD. Am. J. Med. Genet. B Neuropsychiatr. Genet. 147B, 397–402. 10.1002/ajmg.b.30595 17886261

[B13] Bowles B. Crupi C. Mirsattari S. M. Pigott S. E. Parrent A. G. Pruessner J. C.. (2007). Impaired familiarity with preserved recollection after anterior temporal-lobe resection that spares the hippocampus. Proc. Natl. Acad. Sci. U S A 104, 16382–16387. 10.1073/pnas.0705273104 17905870 PMC1995093

[B14] Breedlove S. M. Rosenzweig M. R. Watson N. V. (2010). Biological Psychology/An Introduction to Behavioral, Cognitive and Clinical Neuroscience. 6th Edn. Sunderland, MA: Sinauer Associates.

[B15] Brookes K. J. Mill J. Guindalini C. Curran S. Xu X. Knight J.. (2006). A common haplotype of the dopamine transporter gene associated with attention-deficit/hyperactivity disorder and interacting with maternal use of alcohol during pregnancy. Arch. Gen. Psychiatry 63, 74–81. 10.1001/archpsyc.63.1.74 16389200

[B16] Brookes K. J. Neale B. M. Sugden K. Khan N. Asherson P. D’Souza U. M. (2007). Relationship between VNTR polymorphisms of the human dopamine transporter gene and expression in post-mortem midbrain tissue. Am. J. Med. Genet. B Neuropsychiatr. Genet. 144B, 1070–1078. 10.1002/ajmg.b.30572 17579365

[B18] Bunzeck N. Doeller C. F. Dolan R. J. Duzel E. (2012). Contextual interaction between novelty and reward processing within the mesolimbic system. Hum. Brain Mapp. 33, 1309–1324. 10.1002/hbm.21288 21520353 PMC3498733

[B17] Bunzeck N. Düzel E. (2006). Absolute coding of stimulus novelty in the human substantia nigra/VTA. Neuron 51, 369–379. 10.1016/j.neuron.2006.06.021 16880131

[B19] Bunzeck N. Guitart-Masip M. Dolan R. J. Duzel E. (2014). Pharmacological dissociation of novelty responses in the human brain. Cereb. Cortex 24, 1351–1360. 10.1093/cercor/bhs420 23307638 PMC3977623

[B20] Bunzeck N. Schutze H. Duzel E. (2006). Category-specific organization of prefrontal response-facilitation during priming. Neuropsychologia 44, 1765–1776. 10.1016/j.neuropsychologia.2006.03.019 16701731

[B21] Callan D. E. Schweighofer N. (2008). Positive and negative modulation of word learning by reward anticipation. Hum. Brain Mapp. 29, 237–249. 10.1002/hbm.20383 17390317 PMC6870695

[B22] Chavarría-Siles I. Contreras-Rojas J. Hare E. Walss-Bass C. Quezada P. Dassori A.. (2008). Cannabinoid receptor 1 gene (CNR1) and susceptibility to a quantitative phenotype for hebephrenic schizophrenia. Am. J. Med. Genet. B Neuropsychiatr. Genet. 147B, 279–284. 10.1002/ajmg.b.30592 18186055

[B23] Cheon K.-A. Ryu Y.-H. Kim J.-W. Cho D.-Y. (2005). The homozygosity for 10-repeat allele at dopamine transporter gene and dopamine transporter density in Korean children with attention deficit hyperactivity disorder: relating to treatment response to methylphenidate. Eur. Neuropsychopharmacol. 15, 95–101. 10.1016/j.euroneuro.2004.06.004 15572278

[B24] Chib V. S. De Martino B. Shimojo S. O’Doherty J. P. (2012). Neural mechanisms underlying paradoxical performance for monetary incentives are driven by loss aversion. Neuron 74, 582–594. 10.1016/j.neuron.2012.02.038 22578508 PMC3437564

[B25] Clos M. Bunzeck N. Sommer T. (2019a). Dopamine enhances item novelty detection *via* hippocampal and associative recall *via* left lateral prefrontal cortex mechanisms. J. Neurosci. 39, 7920–7933. 10.1523/JNEUROSCI.0495-19.2019 31405927 PMC6774414

[B26] Clos M. Bunzeck N. Sommer T. (2019b). Dopamine is a double-edged sword: dopaminergic modulation enhances memory retrieval performance but impairs metacognition. Neuropsychopharmacology 44, 555–563. 10.1038/s41386-018-0246-y 30356095 PMC6333779

[B27] Comings D. E. (1998). Polygenic inheritance and micro/minisatellites. Mol. Psychiatry 3, 21–31. 10.1038/sj.mp.4000289 9491809

[B28] Congdon E. Lesch K. P. Canli T. (2008). Analysis of DRD4 and DAT polymorphisms and behavioral inhibition in healthy adults: implications for impulsivity. Am. J. Med. Genet. B Neuropsychiatr. Genet. 147B, 27–32. 10.1002/ajmg.b.30557 17525955

[B29] Dawson E. (1995). Identification of a polymorphic triplet repeat marker for the brain cannabinoid receptor gene: use in linkage and association studies. Psychiatr. Genet. 5:850.

[B30] Dayan P. (2012). Twenty-five lessons from computational neuromodulation. Neuron 76, 240–256. 10.1016/j.neuron.2012.09.027 23040818

[B39] de Fonseca F. R. Gorriti M. A. Bilbao A. Escuredo L. García-segura L. M. Piomelli D.. (2001). Role of the endogenous cannabinoid system as a modulator of dopamine transmission: implications for Parkinson’s disease and schizophrenia. Neurotox. Res. 3, 23–35. 10.1007/BF03033228 15111259

[B31] Diana R. A. Yonelinas A. P. Ranganath C. (2007). Imaging recollection and familiarity in the medial temporal lobe: a three-component model. Trends Cogn. Sci. 11, 379–386. 10.1016/j.tics.2007.08.001 17707683

[B32] Dreber A. Apicella C. L. Eisenberg D. T. A. Garcia J. R. Zamore R. S. Lum J. K.. (2009). The 7R polymorphism in the dopamine receptor D4 gene (DRD4) is associated with financial risk taking in men. Evol. Hum. Behav. 30, 85–92. 10.1016/j.evolhumbehav.2008.11.00110803661

[B33] Düzel E. Bunzeck N. Guitart-Masip M. Wittmann B. Schott B. H. Tobler P. N. (2009). Functional imaging of the human dopaminergic midbrain. Trends Neurosci. 32, 321–328. 10.1016/j.tins.2009.02.005 19446348

[B34] Ehlers C. L. Slutske W. S. Lind P. A. Wilhelmsen K. C. (2007). Association between single nucleotide polymorphisms in the cannabinoid receptor gene (CNR1) and impulsivity in southwest California Indians. Twin Res. Hum. Genet. 10, 805–811. 10.1375/twin.10.6.805 18179391

[B35] Eisenberg D. MacKillop J. Modi M. Beauchemin J. Dang D. Lisman S.. (2007). Examining impulsivity as an endophenotype using a behavioral approach: a DRD2 TaqI A and DRD4 48-bp VNTR association study. Behav. Brain Funct. 3:2. 10.1186/1744-9081-3-2 17214892 PMC1781951

[B36] Fageera W. Grizenko N. Sengupta S. M. Schmitz N. Joober R. (2020). *COMT* by *DRD3* epistatic interaction in modulating behaviors in children with ADHD: a pharmaco-dynamic behavioral approach. J. Atten. Disord. [Epub ahead of print]. 10.1177/1087054720934191 32564645

[B37] Faraone S. V. Biederman J. Weiffenbach B. Keith T. Chu M. P. Weaver A.. (1999). Dopamine D4 gene 7-repeat allele and attention deficit hyperactivity disorder. Am. J. Psychiatry 156, 768–770. 10.1176/ajp.156.5.768 10327912

[B38] Fiorillo C. D. Tobler P. N. Schultz W. (2003). Discrete coding of reward probability and uncertainty by dopamine neurons. Science 299, 1898–1902. 10.1126/science.1077349 12649484

[B40] Froehlich T. E. Lanphear B. P. Dietrich K. N. Cory-Slechta D. A. Wang N. Kahn R. S. (2007). Interactive effects of a DRD4 polymorphism, lead, and sex on executive functions in children. Biol. Psychiatry 62, 243–249. 10.1016/j.biopsych.2006.09.039 17239353

[B41] Gorlick M. A. Worthy D. A. Knopik V. S. McGeary J. E. Beevers C. G. Maddox W. T. (2015). DRD4 long allele carriers show heightened attention to high-priority items relative to low-priority items. J. Cogn. Neurosci. 27, 509–521. 10.1162/jocn_a_00724 25244120 PMC4312507

[B42] Graffelman J. Weir B. S. (2018). Multi-allelic exact tests for Hardy–Weinberg equilibrium that account for gender. Mol. Ecol. Resour. 18, 461–473. 10.1111/1755-0998.12748 29288525 PMC5969302

[B43] Gruber M. J. Otten L. J. (2010). Voluntary control over prestimulus activity related to encoding. J. Neurosci. 30, 9793–9800. 10.1523/JNEUROSCI.0915-10.2010 20660262 PMC2929460

[B44] Guindalini C. Howard M. Haddley K. Laranjeira R. Collier D. Ammar N.. (2006). A dopamine transporter gene functional variant associated with cocaine abuse in a Brazilian sample. Proc. Natl. Acad. Sci. U S A 103, 4552–4557. 10.1073/pnas.0504789103 16537431 PMC1450209

[B45] Guitart-Masip M. Fuentemilla L. Bach D. R. Huys Q. J. Dayan P. Dolan R. J.. (2011). Action dominates valence in anticipatory representations in the human striatum and dopaminergic midbrain. J. Neurosci. 31, 7867–7875. 10.1523/jneurosci.6376-10.2011 21613500 PMC3109549

[B46] Haaker J. Lonsdorf T. B. Schümann D. Bunzeck N. Peters J. Sommer T.. (2017). Where there is smoke there is fear—impaired contextual inhibition of conditioned fear in smokers. Neuropsychopharmacology 42, 1640–1646. 10.25148/etd.fi13101565 28120933 PMC5518897

[B47] Haaker J. Lonsdorf T. B. Schümann D. Menz M. Brassen S. Bunzeck N.. (2015). Deficient inhibitory processing in trait anxiety: evidence from context-dependent fear learning, extinction recall and renewal. Biol. Psychol. 111, 65–72. 10.1016/j.biopsycho.2015.07.010 26219601

[B48] Heinz A. Goldman D. Jones D. W. Palmour R. Hommer D. Gorey J. G.. (2000). Genotype influences *in vivo* dopamine transporter availability in human striatum. Neuropsychopharmacology 22, 133–139. 10.1016/S0893-133X(99)00099-8 10649826

[B49] Jacobsen L. K. Staley J. K. Zoghbi S. S. Seibyl J. P. Kosten T. R. Innis R. B.. (2000). Prediction of dopamine transporter binding availability by genotype: a preliminary report. Am. J. Psychiatry 157, 1700–1703. 10.1176/appi.ajp.157.10.1700 11007732

[B50] Kalisch R. Gerlicher A. M. V. Duvarci S. (2019). A dopaminergic basis for fear extinction. Trends Cogn. Sci. 23, 274–277. 10.1016/j.tics.2019.01.013 30803871

[B51] Knutson B. Adams C. M. Fong G. W. Hommer D. (2001). Anticipation of increasing monetary reward selectively recruits nucleus accumbens. J. Neurosci. 21:RC159. 10.1523/JNEUROSCI.21-16-j0002.2001 11459880 PMC6763187

[B52] Krebs R. M. Schott B. H. Schutze H. Duzel E. (2009). The novelty exploration bonus and its attentional modulation. Neuropsychologia 47, 2272–2281. 10.1016/j.neuropsychologia.2009.01.015 19524091

[B53] Kuhbandner C. Aslan A. Emmerdinger K. Murayama K. (2016). Providing extrinsic reward for test performance undermines long-term memory acquisition. Front. Psychol. 7:79. 10.3389/fpsyg.2016.00079 26869978 PMC4740952

[B54] Kuhnen C. M. Chiao J. Y. (2009). Genetic determinants of financial risk taking. PLoS One 4:e4362. 10.1371/journal.pone.0004362 19209222 PMC2634960

[B55] LaHoste G. J. Swanson J. M. Wigal S. B. Glabe C. Wigal T. King N.. (1996). Dopamine D4 receptor gene polymorphism is associated with attention deficit hyperactivity disorder. Mol. Psychiatry 1, 121–124. 9118321

[B56] Lara A. H. Wallis J. D. (2015). The role of prefrontal cortex in working memory: a mini review. Front. Syst. Neurosci. 9:173. 10.3389/fnsys.2015.00173 26733825 PMC4683174

[B57] Li S.-C. Papenberg G. Nagel I. E. Preuschhof C. Schröder J. Nietfeld W.. (2013). Aging magnifies the effects of dopamine transporter and D2 receptor genes on backward serial memory. Neurobiol. Aging 34:358.358.e1–358.e10. 10.1016/j.neurobiolaging.2012.08.001 22939506

[B58] Lichter J. B. Barr C. L. Kennedy J. L. Tol V. H.m H. Kidd K. K.. (1993). A hypervariable segment in the human dopamine receptor D4 (DRD4) gene. Hum. Mol. Genet. 2, 767–773. 10.1093/hmg/2.6.767 8353495

[B59] Lisman J. E. Grace A. A. (2005). The hippocampal-VTA loop: controlling the entry of information into long-term memory. Neuron 46, 703–713. 10.1016/j.neuron.2005.05.002 15924857

[B60] Lisman J. Grace A. A. Duzel E. (2011). A neoHebbian framework for episodic memory; role of dopamine-dependent late LTP. Trends Neurosci. 34, 536–547. 10.1016/j.tins.2011.07.006 21851992 PMC3183413

[B61] Lonsdorf T. B. Haaker J. Schümann D. Sommer T. Bayer J. Brassen S.. (2015). Sex differences in conditioned stimulus discrimination during context-dependent fear learning and its retrieval in humans: the role of biological sex, contraceptives and menstrual cycle phases. J. Psychiatry Neurosci. 40, 368–375. 10.1503/140336 26107163 PMC4622633

[B62] Loo S. K. Rich E. C. Ishii J. McGough J. McCracken J. Nelson S.. (2008). Cognitive functioning in affected sibling pairs with ADHD: familial clustering and dopamine genes. J. Child Psychol. Psychiatry 49, 950–957. 10.1111/j.1469-7610.2008.01928.x 18665883

[B63] Loureiro M. Renard J. Zunder J. Laviolette S. R. (2015). Hippocampal cannabinoid transmission modulates dopamine neuron activity: impact on rewarding memory formation and social interaction. Neuropsychopharmacology 40, 1436–1447. 10.1038/npp.2014.329 25510937 PMC4397402

[B64] Markett S. A. Montag C. Reuter M. (2009). The association between dopamine DRD2 polymorphisms and working memory capacity is modulated by a functional polymorphism on the nicotinic receptor gene CHRNA4. J. Cogn. Neurosci. 22, 1944–1954. 10.1162/jocn.2009.21354 19803686

[B65] Martin C. B. McLean D. A. O’Neil E. B. Köhler S. (2013). Distinct familiarity-based response patterns for faces and buildings in perirhinal and parahippocampal cortex. J. Neurosci. 33, 10915–10923. 10.1523/JNEUROSCI.0126-13.2013 23804111 PMC6618503

[B66] Martínez-Gras I. Hoenicka J. Ponce G. Rodríguez-Jiménez R. Jiménez-Arriero M. A. Pérez-Hernandez E.. (2006). (AAT)*n* repeat in the cannabinoid receptor gene, CNR1: association with schizophrenia in a Spanish population. Eur. Arch. Psychiatry Clin. Neurosci. 256, 437–441. 10.1007/s00406-006-0665-3 16788767

[B67] Mata R. Hau R. Papassotiropoulos A. Hertwig R. (2012). DAT1 polymorphism is associated with risk taking in the balloon analogue risk task (BART). PLoS One 7:e39135. 10.1371/journal.pone.0039135 22723947 PMC3377600

[B68] Matsuda L. A. Lolait S. J. Brownstein M. J. Young A. C. Bronner T. I. (1990). Structure of a cannabinoid receptor and functional expression of the cloned cDNA. Nature 346, 561’564. 10.1038/346561a0 2165569

[B69] Miller E. K. Lundqvist M. Bastos A. M. (2018). Working memory 2.0. Neuron 100, 463–475. 10.1016/j.neuron.2018.09.023 30359609 PMC8112390

[B70] Mitchell R. J. Howlett S. Earl L. White N. G. McComb J. Schanfield M. S.. (2000). Distribution of the 3′VNTR polymorphism in the human dopamine transporter gene in world populations. Hum. Biol. 72, 295–304. Available online at: https://pubmed.ncbi.nlm.nih.gov/10803661/. 10803661

[B71] Mobbs D. Hassabis D. Seymour B. Marchant J. L. Weiskopf N. Dolan R. J.. (2009). Choking on the money: reward-based performance decrements are associated with midbrain activity. Psychol. Sci. 20, 955–962. 10.1111/j.1467-9280.2009.02399.x 19594859 PMC2931754

[B72] Niv Y. Daw N. D. Joel D. Dayan P. (2007). Tonic dopamine: opportunity costs and the control of response vigor. Psychopharmacology 191, 507–520. 10.1007/s00213-006-0502-4 17031711

[B73] O’Gara C. Stapleton J. Sutherland G. Guindalini C. Neale B. Breen G.. (2007). Dopamine transporter polymorphisms are associated with short-term response to smoking cessation treatment. Pharmacogenet. Genomics 17, 61–67. 10.1097/01.fpc.0000236328.18928.4c 17264803

[B74] Paloyelis Y. Asherson P. Mehta M. A. Faraone S. V. Kuntsi J. (2010). DAT1 and COMT effects on delay discounting and trait impulsivity in male adolescents with attention deficit/hyperactivity disorder and healthy controls. Neuropsychopharmacology 35, 2414–2426. 10.1038/npp.2010.124 20736997 PMC2955909

[B75] Papenberg G. Bäckman L. Nagel I. E. Nietfeld W. Schröder J. Bertram L.. (2013). Dopaminergic gene polymorphisms affect long-term forgetting in old age: further support for the magnification hypothesis. J. Cogn. Neurosci. 25, 571–579. 10.1162/jocn_a_00359 23363412

[B76] Persson N. Persson J. Lavebratt C. Fischer H. (2017). Effects of DARPP-32 genetic variation on prefrontal cortex volume and episodic memory performance. Front. Neurosci. 11:244. 10.3389/fnins.2017.00244 28553197 PMC5425487

[B77] Pessiglione M. Schmidt L. Draganski B. Kalisch R. Lau H. Dolan R. J.. (2007). How the brain translates money into force: a neuroimaging study of subliminal motivation. Science 316, 904–906. 10.1126/science.1140459 17431137 PMC2631941

[B78] Ponce G. Hoenicka J. Rubio G. Ampuero I. Jiménez-Arriero M. A. Rodríguez-Jiménez R.. (2003). Association between cannabinoid receptor gene (CNR1) and childhood attention deficit/hyperactivity disorder in Spanish male alcoholic patients. Mol. Psychiatry 8, 466–467. 10.1038/sj.mp.4001278 12808424

[B79] Raczka K. A. Mechias M.-L. Gartmann N. Reif A. Deckert J. Pessiglione M.. (2011). Empirical support for an involvement of the mesostriatal dopamine system in human fear extinction. Transl. Psychiatry 1:e12. 10.1038/tp.2011.10 22832428 PMC3309464

[B80] Rajagopal V. M. Rajkumar A. P. Jacob K. S. Jacob M. (2018). Gene-gene interaction between DRD4 and COMT modulates clinical response to clozapine in treatment-resistant schizophrenia. Pharmacogenet. Genomics 28, 31–35. 10.1097/fpc.0000000000000314 29087970

[B81] Ranganath C. Yonelinas A. P. Cohen M. X. Dy C. J. Tom S. M. D’Esposito M. (2004). Dissociable correlates of recollection and familiarity within the medial temporal lobes. Neuropsychologia 42, 2–13. 10.1016/j.neuropsychologia.2003.07.006 14615072

[B82] Richter A. Barman A. Wüstenberg T. Soch J. Schanze D. Deibele A.. (2017). Behavioral and neural manifestations of reward memory in carriers of low-expressing versus high-expressing genetic variants of the Dopamine D2 receptor. Front. Psychol. 8:654. 10.3389/fpsyg.2017.00654 28507526 PMC5410587

[B83] Rossato J. I. Bevilaqua L. R. M. Izquierdo I. Medina J. H. Cammarota M. (2009). Dopamine controls persistence of long-term memory storage. Science 325, 1017–1020. 10.1126/science.1172545 19696353

[B84] Roussos P. Giakoumaki S. G. Bitsios P. (2009). Cognitive and emotional processing in high novelty seeking associated with the L-DRD4 genotype. Neuropsychologia 47, 1654–1659. 10.1016/j.neuropsychologia.2009.02.005 19397860

[B85] Ruiz-Contreras A. E. Carrillo-Sánchez K. Gómez-López N. Vadillo-Ortega F. Hernández-Morales S. Carnevale-Cantoni A.. (2013). Working memory performance in young adults is associated to the AAT_n_ polymorphism of the CNR1 gene. Behav. Brain Res. 236, 62–66. 10.1016/j.bbr.2012.08.031 22944513

[B86] Ruiz-Contreras A. E. Delgado-Herrera M. García-Vaca P. A. Almeida-Rosas G. A. Soria-Rodríguez G. Soriano-Bautista A.. (2011). Involvement of the AAT*n* polymorphism of the CNR1 gene in the efficiency of procedural learning in humans. Neurosci. Lett. 494, 202–206. 10.1016/j.neulet.2011.03.013 21396980

[B87] Sauvage M. M. Fortin N. J. Owens C. B. Yonelinas A. P. Eichenbaum H. (2008). Recognition memory: opposite effects of hippocampal damage on recollection and familiarity. Nat. Neurosci. 11, 16–18. 10.1038/nn2016 18037884 PMC4053160

[B88] Schandry R. (2011). Biologische Psychologie/[mit Online-Materialien]. 3., vollst. überarb. Aufl. Weinheim: Beltz.

[B89] Schinka J. A. Letsch E. A. Crawford F. C. (2002). DRD4 and novelty seeking: results of meta-analyses. Am. J. Med. Genet. 114, 643–648. 10.1002/ajmg.10649 12210280

[B90] Schomaker J. Wittmann B. C. (2017). Memory performance for everyday motivational and neutral objects is dissociable from attention. Front. Behav. Neurosci. 11:121. 10.3389/fnbeh.2017.00121 28694774 PMC5483478

[B91] Schott B. H. Seidenbecher C. I. Fenker D. B. Lauer C. J. Bunzeck N. Bernstein H.-G.. (2006). The dopaminergic midbrain participates in human episodic memory formation: evidence from genetic imaging. J. Neurosci. 26, 1407–1417. 10.1523/JNEUROSCI.3463-05.2006 16452664 PMC6675495

[B93] Schultz W. Dayan P. Montague P. R. (1997). A neural substrate of prediction and reward. Science 275, 1593–1599. 10.1126/science.275.5306.1593 9054347

[B92] Schuck N. W. Frensch P. A. Schjeide B.-M. M. Schröder J. Bertram L. Li S.-C. (2013). Effects of aging and dopamine genotypes on the emergence of explicit memory during sequence learning. Neuropsychologia 51, 2757–2769. 10.1016/j.neuropsychologia.2013.09.009 24035787

[B94] Schümann D. Bayer J. Talmi D. Sommer T. (2018). Dissociation of immediate and delayed effects of emotional arousal on episodic memory. Neurobiol. Learn. Mem. 148, 11–19. 10.1016/j.nlm.2017.12.007 29289675

[B95] Sharifian F. Contier O. Preuschhof C. Pollmann S. (2017). Reward modulation of contextual cueing: repeated context overshadows repeated target location. Atten. Percept. Psychophys. 79, 1871–1877. 10.3758/s13414-017-1397-3 28785966 PMC5603623

[B96] Shigemune Y. Abe N. Suzuki M. Ueno A. Mori E. Tashiro M.. (2010). Effects of emotion and reward motivation on neural correlates of episodic memory encoding: a PET study. Neurosci. Res. 67, 72–79. 10.1016/j.neures.2010.01.003 20079775

[B97] Simon J. R. Stollstorff M. Westbay L. C. Vaidya C. J. Howard J. H. Howard D. V. (2011). Dopamine transporter genotype predicts implicit sequence learning. Behav. Brain Res. 216, 452–457. 10.1016/j.bbr.2010.08.043 20817043 PMC2975813

[B98] Simpson J. Vetuz G. Wilson M. Brookes K. J. Kent L. (2010). The DRD4 receptor Exon 3 VNTR and 5′ SNP variants and mRNA expression in human post-mortem brain tissue. Am. J. Med. Genet. B Neuropsychiatr. Genet. 153B, 1228–1233. 10.1002/ajmg.b.31084 20468066

[B99] Simsek M. Al-Sharbati M. Al-Adawi S. Lawatia K. (2006). The VNTR polymorphism in the human dopamine transporter gene: improved detection and absence of association of VNTR alleles with attention-deficit hyperactivity disorder. Genet. Test. 10, 31–34. 10.1089/gte.2006.10.31 16545000

[B100] Singer A. C. Frank L. M. (2009). Rewarded outcomes enhance reactivation of experience in the hippocampus. Neuron 64, 910–921. 10.1016/j.neuron.2009.11.016 20064396 PMC2807414

[B101] Smirnova M. Mitushkina N. Suhovskaya O. Imyanitov E. (2011). Association between dopamine transporter, monoamine oxidases genotypes and tobacco smoking. Eur. Respir. J. 38:p1105. Available online at: https://erj.ersjournals.com/content/38/Suppl_55/p1105.

[B102] Squire L. R. Dede A. J. O. (2015). Conscious and unconscious memory systems. Cold Spring Harb. Perspect. Biol. 7:a021667. 10.1101/cshperspect.a021667 25731765 PMC4355270

[B103] Squire L. R. Stark C. E. L. Clark R. E. (2004). The medial temporal lobe. Annu. Rev. Neurosci. 27, 279–306. 10.1146/annurev.neuro.27.070203.144130 15217334

[B104] Steiger T. K. Bunzeck N. (2017). Reward dependent invigoration relates to theta oscillations and is predicted by dopaminergic midbrain integrity in healthy elderly. Front. Aging Neurosci. 9:1. 10.3389/fnagi.2017.00001 28174533 PMC5258705

[B105] Szekely A. Balota D. A. Duchek J. M. Nemoda Z. Vereczkei A. Sasvari-Szekely M. (2011). Genetic factors of reaction time performance: DRD4 7-repeat allele associated with slower responses. Genes Brain Behav. 10, 129–136. 10.1111/j.1601-183x.2010.00645.x 20807239

[B106] Tobler P. N. Fiorillo C. D. Schultz W. (2005). Adaptive coding of reward value by dopamine neurons. Science 307, 1642–1645. 10.1126/science.1105370 15761155

[B107] Ujike H. Takaki M. Nakata K. Tanaka Y. Takeda T. Fujiwara Y.. (2002). CNR1, central cannabinoid receptor gene, associated with susceptibility to hebephrenic schizophrenia. Mol. Psychiatry 7, 515–518. 10.1038/sj.mp.4001029 12082570

[B108] van Dyck C. H. Malison R. T. Jacobsen L. K. Seibyl J. P. Staley J. K. Laruelle M.. (2005). Increased dopamine transporter availability associated with the 9-repeat allele of the SLC6A3 gene. J. Nucl. Med. 46, 745–751. Available online at: http://jnm.snmjournals.org/content/46/5/745.long. 15872345

[B109] Vann S. D. Tsivilis D. Denby C. E. Quamme J. R. Yonelinas A. P. Aggleton J. P.. (2009). Impaired recollection but spared familiarity in patients with extended hippocampal system damage revealed by 3 convergent methods. Proc. Natl. Acad. Sci. U S A 106, 5442–5447. 10.1073/pnas.0812097106 19289844 PMC2664061

[B110] VanNess S. H. Owens M. J. Kilts C. D. (2005). The variable number of tandem repeats element in DAT1 regulates *in vitro* dopamine transporter density. BMC Genet. 6:55. 10.1186/1471-2156-6-55 16309561 PMC1325255

[B111] Varga G. Szekely A. Antal P. Sarkozy P. Nemoda Z. Demetrovics Z.. (2012). Additive effects of serotonergic and dopaminergic polymorphisms on trait impulsivity. Am. J. Med. Genet. B Neuropsychiatr. Genet. 159B, 281–288. 10.1002/ajmg.b.32025 22259185

[B112] Wells T. T. Beevers C. G. Knopik V. S. McGeary J. E. (2013). Dopamine D4 receptor gene variation is associated with context-dependent attention for emotion stimuli. Int. J. Neuropsychopharmacol. 16, 525–534. 10.1017/s1461145712000478 22607734 PMC3799761

[B113] Wise R. A. (1982). Neuroleptics and operant behavior: the anhedonia hypothesis. Behav. Brain Sci. 5, 39–53. 10.1017/s0140525x00010372

[B114] Wittmann B. C. Schott B. H. Guderian S. Frey J. U. Heinze H.-J. Düzel E. (2005). Reward-related FMRI activation of dopaminergic midbrain is associated with enhanced hippocampus-dependent long-term memory formation. Neuron 45, 459–467. 10.1016/j.neuron.2005.01.010 15694331

[B115] Wittmann B. C. Tan G. C. Lisman J. E. Dolan R. J. Düzel E. (2013). DAT genotype modulates striatal processing and long-term memory for items associated with reward and punishment. Neuropsychologia 51, 2184–2193. 10.1016/j.neuropsychologia.2013.07.018 23911780 PMC3809516

[B116] Yacubian J. Sommer T. Schroeder K. Gläscher J. Kalisch R. Leuenberger B.. (2007). Gene-gene interaction associated with neural reward sensitivity. Proc. Natl. Acad. Sci. U S A 104, 8125–8130. 10.1073/pnas.0702029104 17483451 PMC1864910

[B117] Yonelinas A. P. Aly M. Wang W.-C. Koen J. D. (2010). Recollection and familiarity: examining controversial assumptions and new directions. Hippocampus 20, 1178–1194. 10.1002/hipo.20864 20848606 PMC4251874

[B118] Yonelinas A. P. Dobbins I. Szymanski M. D. Dhaliwal H. S. King L. (1996). Signal-detection, threshold and dual-process models of recognition memory: ROCs and conscious recollection. Conscious. Cogn. 5, 418–441. 10.1006/ccog.1996.0026 9063609

